# S-Nitrosylated Proteins Involved in Autophagy in *Triticum aestivum* Roots: A Bottom-Up Proteomics Approach and In Silico Predictive Algorithms

**DOI:** 10.3390/life13102024

**Published:** 2023-10-08

**Authors:** Anastasia Mazina, Julia Shumilina, Natalia Gazizova, Egor Repkin, Andrej Frolov, Farida Minibayeva

**Affiliations:** 1 Kazan Institute of Biochemistry and Biophysics, FRC Kazan Scientific Center, Russian Academy of Sciences, 420111 Kazan, Russia; abmazina@gmail.com (A.M.); natgazizova@mail.ru (N.G.); 2 Laboratory of Analytical Biochemistry and Biotechnology, Timiryazev Institute of Plant Physiology, Russian Academy of Sciences, 127276 Moscow, Russia; schumilina.u@yandex.ru (J.S.); frolov@ifr.moscow (A.F.); 3 Centre for Molecular and Cell Technologies, Saint Petersburg State University, Universitetskaya Embankment, 7/9, 199034 Saint Petersburg, Russia; st049553@student.spbu.ru; 4 Open Lab ‘Biomarker’, Kazan (Volga Region) Federal University, 420008 Kazan, Russia

**Keywords:** *Triticum aestivum*, autophagy, nitric oxide, protein S-nitrosylation

## Abstract

Autophagy is a highly conserved catabolic process in eukaryotic cells. Reactive nitrogen species play roles as inductors and signaling molecules of autophagy. A key mechanism of NO-mediated signaling is S-nitrosylation, a post-translational modification (PTM) of proteins at cysteine residues. In the present work, we analyzed the patterns of protein S-nitrosylation during the induction of autophagy in *Triticum aestivum* roots. The accumulation of S-nitrosylated proteins in the cells during autophagy induced with KNO_2_ and antimycin A was visualized using monoclonal antibodies with a Western blot analysis, and proteins were identified using a standard bottom-up proteomics approach. Protein S-nitrosylation is a labile and reversible PTM, and therefore the SNO group can be lost during experimental procedures. A subsequent bioinformatic analysis using predictive algorithms and protein-ligand docking showed that identified proteins possess hypothetical S-nitrosylation sites. Analyzing protein–protein interaction networks enabled us to discover the targets that can directly interact with autophagic proteins, and those that can interact with them indirectly via key multifunctional regulatory proteins. In this study, we show that S-nitrosylation is a key mechanism of NO-mediated regulation of autophagy in wheat roots. A combination of in silico predictive algorithms with a mass spectrometry analysis provides a targeted approach for the identification of S-nitrosylated proteins.

## 1. Introduction

Autophagy, a highly conserved catabolic process, is of great importance in eukaryotes. In plants, the role of autophagy has been shown in onto- and organogenesis, for example, in the formation of aerenchyma and xylem vessels [1], and in the processes of aging and programmed cell death (PCD) [2]. Autophagy is also considered as a defense reaction for survival of an organism in stressful environments [3]. It is now well established that autophagy is a very sensitive process involved in cellular response to almost every stressful condition. Such unfavorable factors as starvation, drought or flooding, low or high temperatures, and infection with pathogens can serve as inducers of autophagy in plants [1]. Activation of autophagic processes under stress is necessary for the efficient breakdown of macromolecules to provide cells with construction blocks and energy substrates [4], as well as the timely removal of oxidized or spent macromolecules and damaged structures [5].

Autophagy is characterized by the appearance of double-membrane vesicles (autophagosomes), whose biogenesis involves de novo formation of a membrane that elongates to sequester cytoplasmic cargo and closes to form an autophagosome, controlled by the sequential activity of multiple autophagy-related (ATG) proteins [6]. Structural and functional decipherment of such ATG proteins has shown that they typically form multi-subunit complexes that work together to coordinate the multiple membrane modeling events involved in autophagosome formation. Plant genomes encode a diversity of ATG orthologs identified in yeasts and mammals. ATG proteins are typically divided on four protein clusters: (1) kinase complex ATG1/ATG13; (2) autophagy-specific phosphatidylinositol (PI) 3-kinase complex (PI3K); (3) ATG9 complex; and (4) ubiquitin-like conjugation system ATG8/ATG12 [6].

At present, it is well established that reactive oxygen species (ROS) and reactive nitrogen species (RNS) are among the main intracellular signal transducers sustaining autophagy [4]. The involvement of ROS and RNS, including nitric oxide (NO), in the regulation of autophagy has been most extensively studied in animal cells where evidence exists for both the induction [3,7] and the suppression [8,9] of autophagy with RNS. In photosynthetic organisms, little information is available about the effects of NO on autophagic processes. Induction of autophagosome formation with increasing NO levels has been shown in the roots of wheat seedlings [10]. In the unicellular alga *Chlamidomonas reinhardii*, exposure to high light induced NO emission and cell death via autophagy, which was confirmed by an increase in ATG8 protein content and activation of other ATG genes [11]. Autophagy was suppressed in the presence of the NO acceptor (cPTIO, 2-4-carboxyphenyl-4,4,5,5-tetramethylimidazoline-1-oxyl-3-oxide). Interestingly, co-treatment of cells with H_2_O_2_ and NO donors induced autophagy and resulted in cell death after 24 h, and this effect was also eliminated using cPTIO. These data suggest that the regulatory role of NO in autophagic processes is largely due to the synergistic effect of ROS and RNS [12]. 

Nitric oxide regulates protein activity through various post-translational modifications (PTMs), such as S-nitrosylation of sulfhydryl (thiol) groups of proteins and oxidative nitrosylation of iron and other metal-containing proteins. S-nitrosylation of proteins is a mechanism involving the covalent attachment of the -NO group to the cysteine thiol in a protein, resulting in the formation of S-nitrosothiol (SNO). Despite the importance of this PTM for cellular biology, in plants, our understanding of the molecular mechanisms underlying S-nitrosylation of proteins remains limited. Current estimates suggest that approximately 70% of the proteome is subject to S-nitrosylation, and most of the S-nitrosylation sites are conserved [13]. This particular protein PTM is known to be a key element of the NO-mediated signal transduction mechanism in many processes, including autophagy [14]. Another NO-mediated modification of proteins is tyrosine nitration, which is an irreversible reaction of a nitrating agent with the tyrosine residue of a target protein [15]. Nitration of oxidoreductase enzymes, such as catalase, ascorbate peroxidase, monodehydroascorbate reductase, superoxide dismutase, and nitrate reductase, has been shown to reduce their activity [16]. It seems likely that an increase in protein tyrosine nitration could be considered as a consequence of nitrosative and oxidative stresses. 

The first article on the regulation of autophagy mediated by protein S-nitrosylation in animal cells was published only in 2011 [8]. Currently, the study of the effects of protein S-nitrosylation on autophagic processes is a hot topic in medical research [7,17,18]. Targeting the pro-death and pro-survival functions of protein S-nitrosylation in autophagy has become a novel therapeutic strategy for treatments of Parkinson’s disease [19], cellular tumorigenesis [20,21], myocardial ischemia [22], and cocaine dependence [23]. One of the targets of S-nitrosylation is the family of cysteine proteases ATG4, which occurs universally in eukaryotic cells. For example, ATG4B undergoes S-nitrosylation at Cys189 and Cys292 when glucose levels increase in human neuronal cells [18]. This modification reduces the efficiency of the proteolytic and delipidative activities of ATG4, resulting in the inhibition of the ability of ATG4B to process ATG8 family precursors and deconjugate ATG8 to PE, and as a consequence, in the reduction in autophagic flux.

For plants, only fragmentary information is available about the regulation of autophagy mediated by protein S-nitrosylation. A recent study demonstrated that in *Arabidopsis thaliana*, hypoxic conditions induce S-nitrosylation of the protein AtGSNOR1 (S-nitrosoglutathione reductase (1)) at the conserved Cys10 residue [24]. This, in turn, changes the conformational structure of the protein and promotes its interaction with AtATG8 via the ATG8-interacting motif (AIM). These protein–protein interactions (PPIs) result in the degradation of AtGSNOR1 via selective autophagy. These data provide direct evidence of a functional link between protein S-nitrosylation and autophagy in plants during stress, particularly in response to hypoxia. Unfortunately, proteins that undergo S-nitrosylation have not been identified in plants exposed to conditions that induce autophagy. Therefore, the aim of the present study was to identify potential protein targets of S-nitrosylation in the cells of wheat roots, in which autophagy was induced by the application of antimycin A, a mitochondrial inhibitor, and KNO_2_, a donor of NO. We previously showed that antimycin A [25] and KNO_2_ [26] efficiently induce autophagy in wheat roots. To identify S-nitrosylated proteins, we applied a complex approach including polyacrylamide gel electrophoresis (PAGE) and immunoblotting with monoclonal antibodies followed by protein identification in visualized electrophoretic zones (bands) using the bottom-up proteomics approach, i.e., tryptic digestion of individual bands with subsequent nanoflow-reversed-phase-high-performance-liquid-chromatography–(tandem)-mass-spectrometry (nanoRP-HPLC-MS and MS/MS). Furthermore, this classical bottom-up proteomics approach was combined with algorithms for prediction of hypothetical S-nitrosylation sites and modelling of the protein–ligand molecular interactions. An in silico analysis of PPIs clearly demonstrated that S-nitrosylation of proteins, as a result of NO signaling, provides an important link between the key metabolic pathways and autophagy.

## 2. Materials and Methods

### 2.1. Plant Material

Seeds of bread wheat (*Triticum aestivum* L.) variety Kazanskaya Yubileinaya were purchased from the Tatar Research Institute of Agriculture, FRC Kazan Scientific Center, Russian Academy of Sciences, Kazan, Russia. Seedlings were grown hydroponically in distilled water for 4 d at 22 °C in a growth chamber with a 12 h light/dark photoperiod at a light intensity of 150 μmol photons m^−^² s^−1^. Intact roots were incubated for 3 h in distilled water (control), 1 μM antimycin A (AA), and 1 mM KNO_2_, and for 15 min in 5 mM S-nitrosoglutathione (GSNO).

### 2.2. Extraction and Immunodetection of S-Nitrosylated Wheat Proteins

Water-soluble proteins were isolated from the root tips by fixing them in liquid nitrogen and homogenizing in a medium containing 50 mM HEPES, pH 7.5; 1 mM EDTA; and a protease inhibitor cocktail (Sigma, Roedermark, Germany). The homogenate was then centrifuged at 12,000× *g* and 4 °C for 10 min. Protein electrophoresis was performed in a 4% polyacrylamide stacking gel at 40 V and in a 10% separating gel at 120 V in a Mini-PROTEAN Tetra Cell chamber (Bio-Rad, Hercules, CA, USA) [27]. The content of water-soluble proteins was determined using Qubit™ (Invitrogen, Waltham, MA, USA), according to the manufacturer’s protocol. In all samples, 30 µg of protein was applied to the lane. Proteins separated electrophoretically were transferred onto polyvinylidene difluoride (PVDF) membranes with semi-dry blotting using an SDS-PAGE Transfer Buffer at 150 mA for 1 h. Membranes were incubated with mouse monoclonal antibodies HY8E12 (ab268288, Abcam, Waltham, MA, USA), which specifically recognize bound forms of S-nitroso-L-cysteine, at a dilution of 1:1000. To visualize S-nitrosylated proteins, blots were incubated with secondary anti-mouse IgG antibodies conjugated with horseradish peroxidase (ab205719, Abcam, Waltham, MA, USA) and then with a chemiluminescent substrate (0.1 M Tris-HCl, luminol, *p*-coumaric acid, and 30% H_2_O_2_), and finally scanned using an imaging system (ChemiDoc MP, BioRad, Hercules, CA, USA). The intensity of chemiluminescence was analyzed using ImageLab 6.1 software (BioRad, Hercules, CA, USA).

### 2.3. In-Gel Trypsin Digestion

To identify S-nitrosylated proteins, electrophoretically separated protein bands, which corresponded to those visualized with immune blots, were subjected to in-gel trypsin digestion according to Bassal et al. [28] with minor modifications. A protein band from the gel was cut into small 1–2-mm-sized pieces and after rinsing with LC-Grade H_2_O, the sample was destained with 30% (*v*/*v*) acetonitrile (ACN) in 100 mmol/L NH_4_HCO_3_ and dried under reduced pressure in a vacuum concentrator (CentriVap Vacuum Concentrator, Labconco, Kansas City, MO, USA) at 4 °C. For reduction of disulfides, 10 mM of dithiothreitol (DTT) in 100 mM NH_4_HCO_3_ was supplemented and the samples were incubated for 15 min at 22 °C under continuous shaking in the dark. Afterwards, the alkylation solution (54 mM iodoacetamide in 100 mM NH_4_HCO_3_) was added, and the samples were incubated under the same conditions. Further, the liquid phase was discarded, 100 µL of the destaining solution (30% (*v*/*v*) ACN in 100 mmol/L NH_4_HCO_3_) was added, and gel pieces were shaken for 10 min (450 rpm, 22 °C). This step was repeated twice. The stock solution of trypsin (0.5 µg/µL) was freshly prepared in 50 mmol/L of the NH_4_HCO_3_ buffer, added to the solutions (with an enzyme-to-protein ratio *w*/*w* of 1:20), and then incubated using continuous shaking (450 rpm, 37 °C, 4 h). After that, an additional portion of the trypsin stock solution (with an enzyme-to-protein ratio *w*/*w* of 1:50) was added and the samples were left shaken overnight (450 rpm, 37 °C). The next day, 70 µL of the extraction solution (4% *v*/*v* trifluoroacetic acid in 30% *v*/*v* ACN) was added to the digest mixture and shaken at 22 °C for 40 min, and then the supernatants were collected. This step was repeated twice, and then samples were dried under the reduced pressure in the vacuum concentrator at 4 °C.

### 2.4. Solid-Phase Extraction

The proteolytic hydrolysates were pre-cleaned with reversed-phase solid-phase extraction (RP-SPE) using the elution scheme of Spiller et al. [29] with minor modifications [30]. The pooled eluate was dried (4 °C) under reduced pressure in a vacuum concentrator.

### 2.5. Nano-LC-MS/MS

Protein hydrolysates were loaded onto an Acclaim PepMap 5 mm Trap Cartridge (Thermo Fisher Scientific, Waltham, MA, USA) and separated on a Bruker FORTY separation column (C18 ReproSil AQ, 40 cm 75 m, 1.9 m, 120 A; Bruker Daltonics, Bremen, Germany) using a nanoElute UHPLC chromatography system (Bruker Daltonics) coupled on-line to a TimsToF Pro quadrupole time-of-flight mass spectrometer (QqTOF-MS) via a CaptiveSpray ion source (Bruker Daltonics). Details of the chromatographic separation method are summarized in Supplementary Information S1 (Tables S1–S3). The UHPLC-QqTOF-MS/MS analysis relied on data-dependent acquisition experiments performed in the positive ion mode, comprising survey TOF-MS scans and dependent MS/MS scans for the most abundant signals with the charge states ranging from 2 to 5 at the acquisition rate of 8 to 32 Hz in the cycle time of 3 s. The mass spectrometer settings are summarized in Table S2.

### 2.6. Data Analysis

The mass spectrometry proteomics data were available via ProteomeXchange with identifier PXD044484 and 10.6019/PXD044484. An analysis of the LC-MS and MS/MS data was accomplished with Peaks Xpro software (Bioinformatics Solutions Inc., Waterloo, ON, Canada) (for the detailed settings, see Supplementary Information S1, Table S3). Identification of peptide sequences and annotation of the proteins relied on the common sequence database of the plants representing *Triticum aestivum* and *Arabidopsis thaliana* species (UniProt database, downloaded on the 28 July 2023). Possible sample contaminations were excluded using the CRAP database of common protein contaminations (https://www.thegpm.org/crap/ accessed on 28 July 2023). The following amino acid modification was included in the search: carbamidomethylation of cysteines (mass increment = 57.0215). The following variable amino acid modifications were included in the search: methionine oxidation (15.9949), N-terminal acetylation (42.0106), asparagine and glutamine deamidation (0.9840), and S-nitrosylation of cysteines (28.9902). The MS1 and MS2 tolerance was 10 ppm and 0.05 Da, respectively. FDR (false discovery rate) correction was employed at the level of 0.02. The proteins were identified with at least two unique peptides. Functional annotation and protein localization relied on the UNIPROT database and employed the UNIPROT categories.

### 2.7. Bioinformatic Analysis of the Availability of S-Nitrosylation Sites of Wheat Proteins

To predict the presence of S-nitrosylation sites, amino acid sequences were selected from the UNIPROT database and analyzed using the programs iSNOPseAAC (app.aporc.org/iSNO-PseAAC/ accessed on 10 August 2022), iSNO-AAPair (app.aporc.org/iSNO-AAPair/ accessed on 10 August 2022), GPS-SNO 1.0 (high specificity—high threshold), and pLMSNOSite (github.com/KCLabMTU/glasnost accessed on 15 July 2023). The programs have different databases of S-nitrosylated proteins and algorithms for internal evaluation of prediction efficiency. Based on the differences in the algorithms and internal bases of these programs, sites that were predicted with at least three programs were identified.

### 2.8. Protein–Ligand Docking and Protein–Protein Interactions

Protein models were constructed in AlphaFold (alphafold.ebi.ac.uk/ accessed on 20 August 2022), and the quality of the constructed models assessed using Procheck, GSNO (ZINC3872731) was selected as a ligand from the ZINC database (zinc.docking.org/ accessed on 1 September 2022). Molecular interactions were predicted using the SwissDock web service (www.swissdock.ch/docking accessed on 10 September 2022), and then the data were analyzed using Chimera 1.14.

To construct PPI networks, STRING v11.5 was applied [31]. The networks included a list of identified proteins, ATG proteins, and predicted functional partners, i.e., proteins that were proposed with the STRING program. We used 13 neighborhood interactors that interact with the introduced proteins. All interactions between proteins were constructed based on previous knowledge in curated databases at a medium level of confidence (sources: textmining, experiments, databases, co-expression, neighborhood, gene fusion; score ≥ 0.4).

### 2.9. Statistical Analysis

Protein immunodetection experiments were performed in six biological replicates. The experiment on the identification of S-nitrosylated proteins was performed using two technical replicates.

## 3. Results

### 3.1. Extraction and Visualization of S-Nitrosylated Proteins during Induction of Autophagy

**Figure 1:**
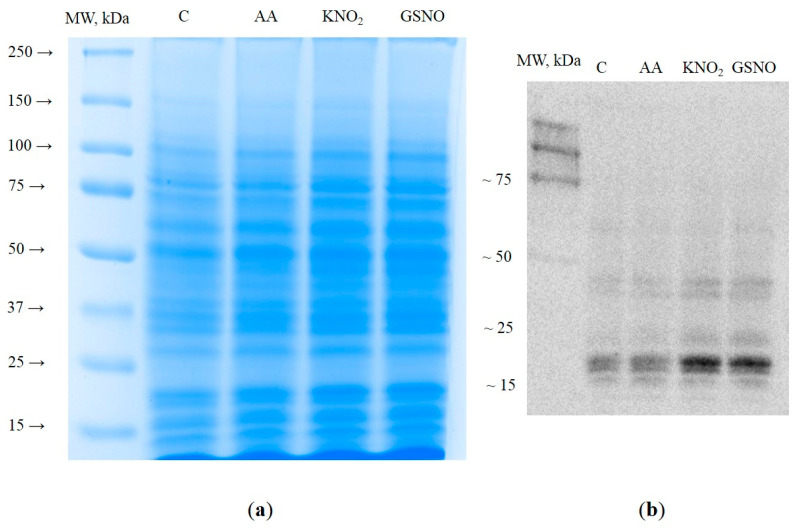
Electropherogram of total water-soluble proteins from wheat roots stained with colloidal Coomassie Brilliant Blue G-250 (**a**). Visualization of S-nitrosylated proteins with Western blot after incubation with mouse monoclonal primary anti-nitroso-*L*-cysteine antibodies and secondary anti-mouse IgG antibodies conjugated with horseradish peroxidase (**b**).

Experimental detection of S-nitrosylated proteins was performed in the water-soluble protein fractions of wheat roots after a 3 h incubation in solutions of 1 µM AA and 1 mM KNO_2_. Roots incubated in 5 mM GSNO were used as a positive control. Determination of the protein concentrations in the obtained isolates revealed extraction yields in the range of 0.201–0.242 mg/μL. In all samples, 30 µg of protein was applied to the lane (Figure 1).

After separation by denaturing PAGE of water-soluble proteins (Figure 1a) and Western blotting on PVDF membranes (Figure 1b), the electrophoretic zones containing S-nitrosylated proteins were visualized with monoclonal primary anti-nitroso-L-cysteine antibodies and secondary peroxidase-conjugated antibodies. It was found that in all treatments, polypeptides (PP) with a molecular mass less than 75 kDa are subjected to S-nitrosylation. After incubation of the roots in KNO_2_ and GSNO solutions, the luminescence intensity of multiple PPs with a molecular mass less than 50 kDa markedly increased. In particular, PPs with molecular weights less than 25 kDa exhibited the most intensive chemiluminescence. Indeed, the luminescence intensity of PPs with a molecular mass less than 50 and 25 kDa in KNO_2_ and GSNO samples was 1.5–1.7 times higher than that in the control (Figure 1b). Next, the blots were matched to the gels stained with colloidal Coomassie Brilliant Blue G-250, and gel bands corresponding to the electrophoretic zones matched to the areas stained with antibodies were cut and subjected to in-gel trypsin digestion for protein identification (Figure S1).

### 3.2. Protein Identification and Search for S-Nitrosylation Sites

A total of 298 proteins were identified with the nanoRP-HPLC-MS/MS analysis of tryptic protein hydrolysates (Table S4). More than half of these proteins (177 species) were present in all experimental groups. All proteins identified in the control were also found in KNO_2_, antimycin A, and GSNO groups.

**Figure 2:**
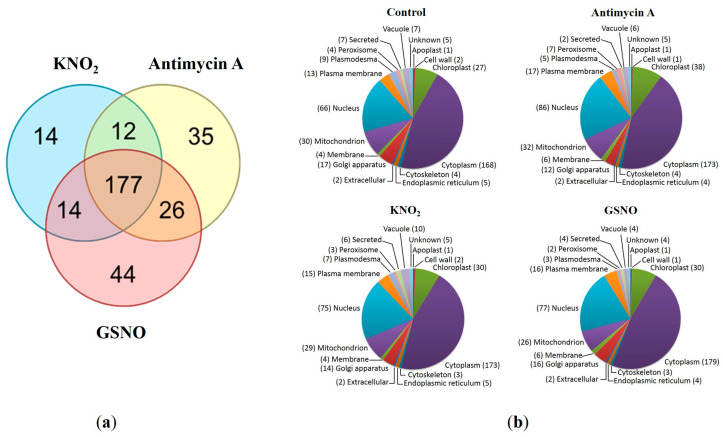
Venn diagram showing the overlapped identified proteins from the samples from the roots treated with KNO_2_, antimycin A, and GSNO. The numbers of the proteins identified with the tandem mass spectra of corresponding peptides in the digests obtained from the protein isolates of different treatments (**a**); prediction of cellular localization of identified proteins from the samples of different treatments using the UNIPROT database and the UNIPROT categories (**b**).

An analysis of the group-specific changes in the root proteome revealed 35 unique proteins in the roots treated with antimycin A, 14 proteins in the roots treated with KNO_2_, and 44 proteins in the roots treated with GSNO (Figure 2a).

Functional annotation of the total protein number identified in all groups with the UNIPROT database and its classification categories showed that most of the proteins are responsible for protein biosynthesis and metabolism, transport, and carbohydrate metabolism (Figure S2 and Table S5). In particular, identified polypeptides included those involved in protein metabolism (40S and 60S ribosome subunits, ATP synthase, and 26S proteasome subunits), protein biosynthesis initiation and elongation factors, heat shock protein (70 kDa), and core histones. In all samples, the proteins were predominantly localized in the cytoplasm, nucleus, mitochondria, and chloroplasts (Figure 2b and Table S6).

**Figure 3:**
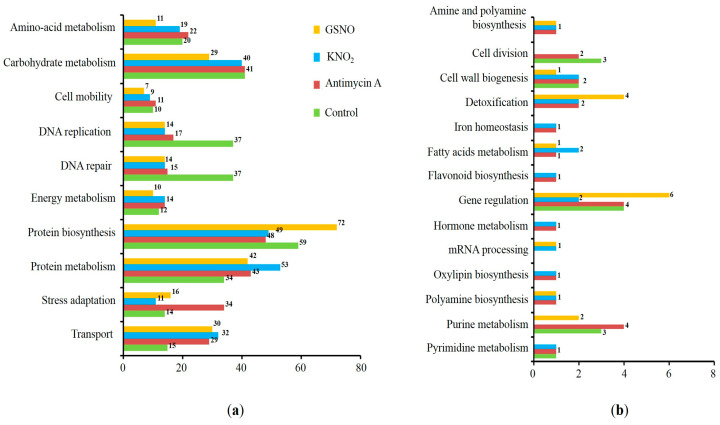
Functional annotation of the total identified proteins. The proteins representing the major (**a**) and minor (**b**) functional categories (i.e., the functional groups with the most and the least represented number of proteins, respectively).

For the convenience of interpretation, regarding the results of functional annotation, we separately considered the proteins representing the major and the minor functional categories (i.e., those with the most and the least represented number of proteins) (see Figure 3a,b, respectively). Thus, the proteins involved in carbohydrate metabolism (aconitate hydratase and triosephosphate isomerase), stress adaptation (2-cys peroxiredoxin and L-ascorbate peroxidase), and transport (ADP, ATP carrier protein 1, and V-type proton ATPase subunit) were also identified among the major functional category groups (Figure 3a). The proteins representing minor functional categories included those involved in such processes as the biosynthesis of flavonoids (tricetin 3′,4′,5′-O-trimethyltransferase), polyamines (N-carbamoylputrescine amidase), oxylipins (putative 12-oxophytodienoate reductase), and pyrimidines (uridine 5′-monophosphate synthase) (Figure 3b). Interestingly, the proteins related to DNA replication and repair were the most abundant in the control group. To make the FDR correction less strict, a search for S-nitrosylated peptides (i.e., those containing the thionitroso group, SNO) relied on the search of the tandem-mass spectrometric data against the fasta file containing the list of only identified proteins. This analysis showed that the SNO group was lost during the sample preparation procedures. As S-nitrosylation is well known to be a highly labile PTM, this scenario seems to be most likely. Indeed, the reaction of thiol nitrosylation is very sensitive to experimental conditions, especially to the presence of reducing agents (e.g., DTT) used in sample preparation, light, etc. Nevertheless, the possibility of S-nitrosylation of identified proteins in planta was appreciated by predicting S-nitrosylation sites.

### 3.3. Prediction of S-Nitrosylation Sites, Localization, and Functional Annotation of S-Nitrosylated Proteins

A further analysis of protein sequences using the iSNOPseAAC, iSNO-AAPair, GPS-SNO 1.0, and pLMSNOSite programs revealed 78 proteins (predicted with three programs; Table S7) and 17 proteins (predicted with all four programs; Table 1), which possess hypothetical S-nitrosylation sites.

**Figure 4:**
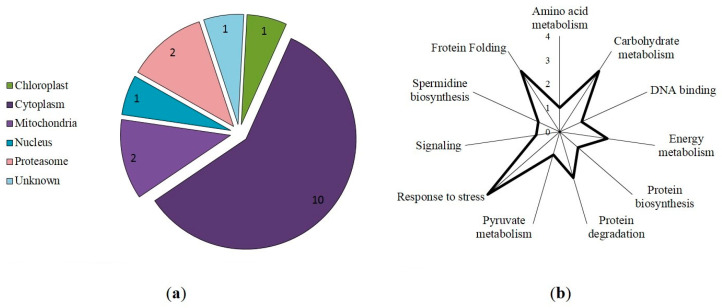
Predicted intracellular localization (**a**) and functional annotation (**b**) of S-nitrosylated proteins in wheat roots treated with antimycin A, KNO_2_, and GSNO from Table 1.

Most of the identified S-nitrosylated proteins were localized in the cytoplasm and mitochondria (Figure 4a). Interestingly, S-nitrosylation sites in the proteins involved in protein biosynthesis, stress response, and carbohydrate metabolism were predicted with three programs (Table S7), while the modification sites in the proteins involved in cytoskeleton function, protein folding, and general stress response (Figure 4b) were predicted with four programs (Table 1).

### 3.4. Protein–Ligand Docking and Protein–Protein Interactions

Protein–ligand docking was performed to computer-simulate possible S-nitrosylation of proteins using GSNO, a biological source of NO [32], as a ligand.

**Figure 5:**
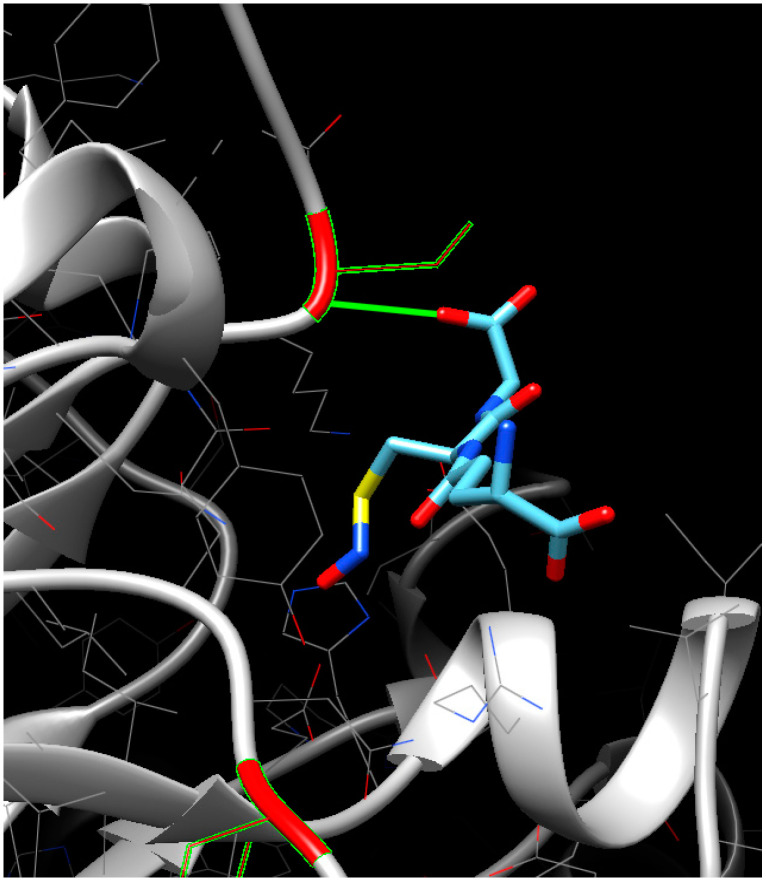
Protein–ligand molecular docking. Interaction (highlighted in green) of cysteine (highlighted in red) of triosophosphate isomerase with GSNO.

Prediction of the molecular interactions of proteins with GSNO using the SwissDock web service and Chimera 1.14 software demonstrated that GSNO interacts with cysteine in triosophosphate isomerase (Figure 5) and mitochondrial ADP/ATP transporter at previously predicted positions of hypothetical S-nitrosylation (Table 1). Interestingly, GSNO interacted with cysteine in spermidine synthase at position 43 that was not predicted with S-nitrosylation site search programs (Table 1). Protein–ligand modelling suggested that for spermidine synthase, this PTM is high-energy-consuming and therefore may be quite rare in a living system. Interestingly, while heat shock proteins and the 14-3-3-like protein GF14 lacked interactions with GSNO, software predicted hypothetical S-nitrosylation sites (Table 1).

To assess possible involvement of the proteins identified with nanoLC-MS/MS and containing prospective S-nitrosylation sites (Section 3.2) in the autophagy, PPI networks were constructed using the STRING online service. For this, we added to the list of identified proteins several autophagic (ATG) proteins involved in four different stages of autophagosome formation: ATG8-conjugation system (ATG8, ATG4, ATG7, and ATG3); ATG12-conjugation system (ATG12, ATG7, ATG10, ATG5, and ATG16); ATG1 protein kinase complex (ATG1, ATG13, ATG17, and ATG29); Vps34 PI3 kinase complex (Vps34, Vps15, ATG14, ATG6, and ATG9); and ATG2-ATG18 complex (ATG2, ATG18, and ATG9). Moreover, we added to this list two further proteins’ target of rapamycin (TOR) kinase and SNF1-related kinase (SnRK1), which are the negative and positive regulators of autophagic processes. To link the proteins identified in this study with the ATG proteins, 13 predicted functional partners proposed with the STRING program were also introduced (Table 2). A network analysis demonstrated that selected proteins have more interactions with each other than could be expected for a random set of proteins. This enrichment indicates that the proteins are potentially biologically related (*p* = 13 × 10^−10^). From an expected number of 364 edges, the number of nodes is 56 (avg. local clustering coefficient is 0.775).

**Figure 6:**
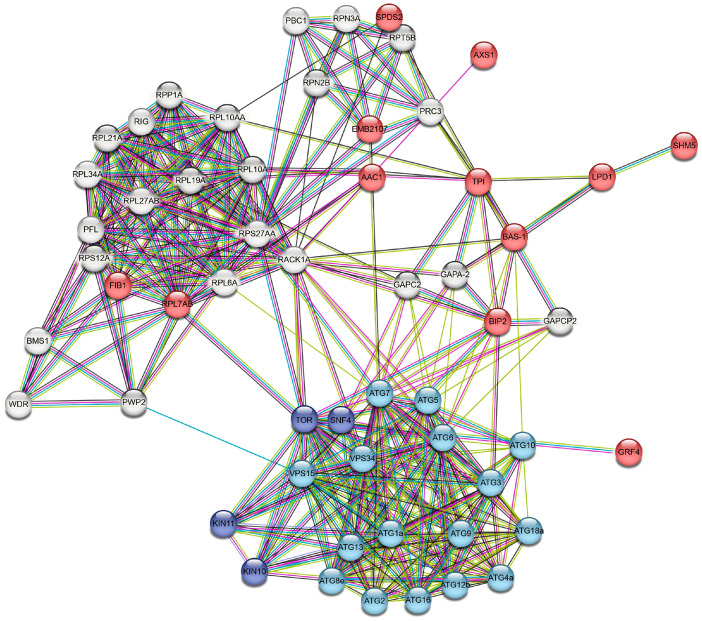
STRING protein–protein interaction network based on the homology to proteins from Arabidopsis thaliana. Proteins marked in red were identified and predicted to have a hypothetical S-nitrosylation site; blue proteins were autophagy-related proteins; navy blue proteins were multifunctional regulatory proteins involved in autophagy; and gray proteins—predicted functional partners proposed with the STRING program. Colored lines between proteins indicate different types of interaction evidence: blue—from curated databases, pink—experimentally determined, yellow—textmining, and black—co-expression.

Importantly, we found possible PPIs between key ATG proteins and proteins identified using LC-MS/MS with predicted S-nitrosylation sites (Figure 6 and Table 2 and Table S9). The following interactions were found: 2-cys peroxiredoxin BAS1 (BAS-1) with ATG10 (combined score of 0.6) and ATG6 (combined score of 0.5); heat shock 70 kDa protein (BIP2) with VPS34 (combined score of 0.7), ATG7, ATG6, ATG3, and ATG5 (combined score of 0.6); 26S proteasome non-ATPase regulatory subunit 12 (EMB2107) with ATG7 (combined score of 0.5); and 14-3-3-like protein (GRF4) with TOR (combined score of 0.6). It is noteworthy that ATG proteins could also interact with proteins identified with nanoLC-MS/MS and could affect their expression (Table S9).

## 4. Discussion

Autophagy is essential for a number of key developmental processes and biotic and abiotic stress responses in plant cells. Although varieties of physiological functions of autophagy are recognized, some of the molecular mechanisms are not fully known yet. Although recent evidence suggests that autophagy in plants is regulated with several PTMs, the information about autophagy regulation with S-nitrosylation is scarce. S-nitrosylation of proteins is a key mechanism of NO-mediated signaling in cells, and it is important to develop an efficient approach to search for S-nitrosylated target proteins. Therefore, in this study, we applied a combination of methods including immunoblotting, protein identification using a standard bottom-up proteomics approach, predictive algorithms, and molecular docking to search for target proteins, i.e., for the potential candidates for S-nitrosylation. While only a few ATG proteins are predicted to be capable of S-nitrosylation, this study identified several S-nitrosylated proteins, which are potentially involved in autophagic flux in wheat roots. Using PPI networks, we found that some of these proteins identified with LC-MS/MS can directly interact with ATG proteins, while others interact with ATG proteins indirectly via key multifunctional regulatory proteins.

Immunoblotting with monoclonal anti-nitroso-*L*-cysteine antibodies revealed the accumulation of S-nitrosylated proteins in the cells of the roots treated with the NO donor KNO_2_ and the mitochondrial inhibitor antimycin A (Figure 1b). The accumulation of S-nitrosylated proteins in the roots following treatment with KNO_2_ is probably a result of the increase in intracellular NO levels that occurs during the reduction of nitrite to NO with nitrite:NO reductase at the plasma membrane [33] and the mitochondrial ETC [34]. Previously, we showed that treatment of wheat roots with the inhibitor of mitochondrial complex III antimycin A induces autophagy, which was confirmed with the accumulation of autophagosomes in the cells [25] and an increase in *ATG* gene expression. Mitochondrial ETC is known as one of the greatest intracellular sources of NO [34]. Disruption of mitochondrial complexes causes an increase in mitochondrial NO levels, which can intensify the process of S-nitrosylation of proteins.

Functional annotation of the identified proteins demonstrated that out of 298 proteins, the most representative group of proteins are involved in protein biosynthesis and metabolism, transport, and carbohydrate metabolism (Figure S2). Among these proteins, 178, which were predicted to contain S-nitrosylation sites in their sequence, are involved in general universal stress response (Figure 4b). The search for PTMs in the total proteome of the organism leads to a large number of single predictions and a relatively tight correction for multiple comparisons (FDR correction) of the probabilities of each of them. In turn, this leads to the loss of relatively low-confidence peptide spectra, which typically include post-translationally modified peptides. To avoid these losses of valuable information, such spectra are typically manually inspected. Indeed, as a result of the low contents of modified peptides in the total hydrolysates, the intensity of the corresponding MS signals is low. Moreover, pronounced ion suppression, which is characteristic for electrospray ionization [35], further reduces signal strength. The low signal intensity of MS1 spectra results in a low intensity of fragment spectra and insufficient representation of individual signals of b- and y-fragment series. In turn, this reduces the probability of identifying the corresponding peptides and Xcorr values obtained in the Sequest search. Such peptides with low probability values do not pass FDR correction and are rejected by the search engine as false positives.

A search for the thionitroso (SNO) group in the identified proteins revealed that this group is lost during sample preparation for a mass spectrometric analysis. The SNO group is known to be highly labile and sensitive to experimental conditions. Indeed, in the absence of immediate chemical derivatization of the nitroso group (e.g., with the biotin switch method), the reverse reaction—denitrosylation—begins to prevail. It leads to the degradation of the modification and the impossibility of its detection [36]. It seems likely that the main reason for the secondary degradation of the modification is the use of the relatively strong reducing agent DTT during sample preparation for the nano-LC-MS/MS analysis [37]. Nevertheless, the presence of S-nitrosylated proteins is confirmed with the interaction of proteins in extracts with monoclonal antibodies for nitroso-*L*-cysteine (Figure 1b), and in silico identification of S-nitrosylation sites in proteins identified with LC-MS/MS. Three programs predict seventy-eight proteins with a S-nitrosylation site, while seventeen proteins are predicted with all four programs (Table 1). Interestingly, among the identified S-nitrosylated proteins, PPs localized in the cytoplasm and mitochondria predominate. Identified proteins with a predicted SNO group include those involved in protein biosynthesis, responses to stressors, carbohydrate metabolism, cytoskeletal functioning, protein folding, and energy metabolism. For example, modeling of triosophosphate isomerase, a glycolytic enzyme involved in carbohydrate metabolism, confirms possible S-nitrosylation at Cys127 (Figure 4). A preliminary analysis using S-nitrosylation site prediction software shows that isoforms of the autophagic proteins ATG4 and ATG16 have hypothetical S-nitrosylation sites (Table S8). This, in turn, may indicate the possibility of S-nitrosylation of ATG proteins.

Using PPI networks, we found proteins such as 14-3-3 proteins (GRF4), heat shock protein 70 kDa (Hsp70 and BIP2), 2-cys peroxiredoxin (BAS-1), and ADP/ATP transporter (AAC1) can directly interact with ATG proteins, while triosephosphate isomerase (TPI), 60S ribosomal protein (RPL7AB), and 26S proteasome non-ATPase regulatory subunit (EMB2107) interact with ATG proteins through intermediary proteins. These proteins can be divided into three groups: (1) proteins that negatively regulate autophagy (14-3-3 protein, heat shock 70 kDa, and ADP/ATP carrier); (2) proteins that can be selectively taken up by autophagosomes (26s proteasome sub. And 60s ribosomal protein); and (3) proteins whose expression depends on the expression of ATG proteins (2-cys peroxiredoxin and triosephosphate isomerase). Negative regulators of autophagy include proteins with diverse effects on autophagy flux. For example, Hsp70 is known to regulate autophagy activity by interacting with p62 protein [38]. However, recent studies showed that Hsp70 that is induced by the stress response can also inhibit autophagy by activating the RAC-alpha serine/threonine-protein kinase (Akt), which in turn phosphorylates and stimulates mTOR. An obvious negative regulator of autophagy is the family of 14-3-3 proteins, which are involved in various, multiple molecular interactions and implicated in subcellular localization, scaffolding, and stability of proteins. The 14-3-3 proteins block the formation of autophagosomes by interacting with phosphorylated ULK1 (ATG1) [39]. Furthermore, in a later stage of autophagosome formation, 14-3-3 proteins can interfere with the activity of hVps34 Beclin-1 (ATG6), thereby inhibiting the autophagy process [40]. Moreover, in plants, the 14-3-3-like protein GRF10 and ATG10 are linked through a product of RNA degradation, namely 2′,3′-cyclic adenosine monophosphate (2′,3′-cAMP) [41]. Accumulation of Br-2′,3′-cAMP/2′,3′-cAMP decreases the expression levels of 14-3-3-like protein but increases the levels of ATG10. Another protein that can stimulate or repress autophagy is the mitochondrial ADP/ATP transporter (AAC), the major transport protein of the inner mitochondrial membrane. This protein exchanges mitochondrial ATP for cytosolic ADP, thereby controlling the production of cellular ATP. It was experimentally shown that in yeast, AAC proteins are involved in the degradation of mitochondria by facilitating permeabilization of mitochondrial membranes and thus triggering PCD [42].

A second regulatory group includes proteins that are involved in selective autophagy, such as selective degradation of ribosomes via ribophagy. Ribosomal assemblies and the abundance of individual ribosomal proteins can be controlled with both the ubiquitin system and autophagy. Interestingly, in human cell lines, it was shown that an increase in NO levels can decrease translational activity and induce ribosome collision, leading to the ribotoxic stress response [43]. Therefore, a surge of NO may be one of the stimuli for the selective removal of ribosomes. Another NO-sensitive system is the 26S proteasome, a multi-subunit proteolytic machine. It has been shown that in endothelial cells, eNOS-derived NO functions as a physiological suppressor of the 26S proteasome in vascular endothelial cells [44]. Notably, nuclear 26S proteasomes can be substrates of autophagy after nitrogen starvation or inactivation. In plants, excess or damaged proteasomes can be degraded in autophagy pathways mediated with signals from the nutrient-responsive kinase ATG1, the ubiquitin subunit, and autophagy receptors, including RPN10. RPN10 acts as a selective autophagy receptor that targets inactive 26S proteasomes through simultaneous interactions with ubiquitylated subunits/targets of proteasomes and lipidated ATG8 lining enveloped autophagic membranes [45].

Positive regulation of autophagy by 2-cys peroxiredoxin (2-CP) is confirmed by the co-expression of transcripts encoding 2-cys peroxiredoxin and ATG in tomato plants exposed to heat stress [46]. Silencing ATG5 or ATG7 increased transcript and protein levels of 2-CP but decreased heat stress tolerance, suggesting that peroxiredoxin plays an important role in stress adaptation of plants through activation of autophagy [47]. Another protein, triosephosphate isomerase, is also involved in the autophagy process. For example, it was shown that airborne PM0.1 nanoparticles (diameter < 100 nm) induce autophagic cell death, which was characterized with the expression of ATG3, 7 and 8 proteins, and triosephosphate isomerase in SH-SY5Y cells [46].

Taken together, our data demonstrate that numerous proteins involved in autophagy in wheat roots are capable of S-nitrosylation. Mechanisms of their involvement in the autophagic machinery are diverse and can include both direct interactions with ATG proteins and indirect interactions with components of signaling transduction pathways, e.g., receptors and protein kinases, resulting in suppression or activation of autophagic flux.

## 5. Conclusions

The rapid activation of autophagy depends largely on PTMs of the proteins that function as molecular switches of autophagic signals. Disruption of autophagy regulation can be detrimental to normal metabolism and lead to pathology; therefore, understanding how PTMs regulate autophagy is extremely important. In this study, we demonstrate that S-nitrosylation is a key mechanism of NO-mediated regulation of autophagy in wheat roots. Detection of the SNO group directly in a mixture of proteins with mass spectrometry is known to be a challenging experimental task. An additional difficulty is that the S-NO bond is sensitive to hydrolysis and unstable, which makes detection of SNO modifications of proteins even more difficult. Nevertheless, the methods of immunoblotting and standard bottom-up proteomics in combination with in silico predictive algorithms allowed us to identify proteins with potential S-nitrosylation sites. Using PPI networks enabled us to find the proteins that can directly interact with ATG proteins, and those that can interact with them indirectly via key multifunctional regulatory proteins. The results obtained in this work can contribute to the development of effective protocols for the identification and analysis of proteins capable of S-nitrosylation. Moreover, our findings expand our understanding of NO-mediated mechanisms of regulation of autophagy and stress response in wheat. Ultimately, this can contribute to the development of effective ways of increasing the stress tolerance of plants, including crops, and enable producers to meet the growing demand for high-quality food and feed products.

## Figures and Tables

**Table 1:** Hypothetical S-nitrosylation sites of identified proteins predicted with iSNOPseAAC, iSNO-AAPair, GPS-SNO 1.0, and pLMSNOSite.

Uniprot ID	Protein	Hypothetical S-Nitrosylation Sites
P46077	14-3-3-like protein GF14 phi	106
Q9FIB6	26S proteasome non-ATPase regulatory subunit 12 homolog A	397
Q9LNU4	26S proteasome non-ATPase regulatory subunit 3 homolog A	141
P80602	2-Cys peroxiredoxin BAS1 chloroplastic (fragment)	64, 185
Q9LZH9	60S ribosomal protein L7a-2	193
P31167	ADP ATP carrier protein 1 mitochondrial	130
Q41629	ADP ATP carrier protein 1 mitochondrial	81, 206
A8MS68	Dihydrolipoyl dehydrogenase 1 chloroplastic	400
Q9S7C0	Heat shock 70 kDa protein 14	268
F4HQD4	Heat shock 70 kDa protein 15	268
Q39043	Heat shock 70 kDa protein BIP2	298
Q9FEF8	rRNA 2′-O-methyltransferase fibrillarin 1	252
Q9SVM4	Serine hydroxymethyltransferase 5	324
O48661	Spermidine synthase 2	43
P48491	Triosephosphate isomerase cytosolic	13, 127
Q9ZUY6	UDP-D-apiose/UDP-D-xylose synthase 1	187
Q9SGE0	UDP-D-apiose/UDP-D-xylose synthase 2	187

**Table 2:** List of the proteins included in the STRING protein–protein interaction network: proteins with hypothetical S-nitrosylation sites, ATG proteins, and 13 predicted functional partners proposed with the STRING program.

Name	Description (UNIPROT)	UNIPROT ID
GRF4	14-3-3-like protein GF14 phi	P46077
EMB2107	26S proteasome non-ATPase regulatory subunit 12 homolog A	Q9FIB6
BAS-1	2-Cys peroxiredoxin BAS1 chloroplastic (fragment)	Q9C5R8
RPL7AB	60S ribosomal protein L7a-2	Q9LZH9
AAC1	ADP ATP carrier protein 1 mitochondrial	P31167
LPD1	Dihydrolipoyl dehydrogenase 1 chloroplastic	A8MS68
BIP2	Heat shock 70 kDa protein BIP2	Q39043
FIB1	rRNA 2′-O-methyltransferase fibrillarin 1	Q9FEF8
SHM5	Serine hydroxymethyltransferase 5	Q9SVM4
SDS2	Spermidine synthase 2	O48661
TPI	Triosephosphate isomerase cytosolic	P48491
AXS1	UDP-D-apiose/UDP-D-xylose synthase 1	Q9ZUY6
ATG1a	Serine/threonine-protein kinase ATG1a	Q94C95
ATG2	Autophagy-related protein 2	F8S296
ATG3	Autophagy-related protein 3	Q0WWQ1
ATG4a	Cysteine protease ATG4a	Q8S929
ATG5	Autophagy protein 5	Q9FFI2
ATG6	Beclin-1-like protein	Q9M367
ATG7	Ubiquitin-like modifier-activating enzyme atg7	Q94CD5
ATG8e	Autophagy-related protein 8e	Q8S926
ATG9	Autophagy-related protein 9	Q8RUS5
ATG10	Ubiquitin-like-conjugating enzyme ATG10	Q8VZ52
ATG12b	Ubiquitin-like protein ATG12B	Q9LVK3
ATG13	Autophagy-related protein 13a	Q9SCK0
ATG16	Autophagy-related protein 16	Q6NNP0
ATG18a	Autophagy-related protein 18a	Q93VB2
TOR	Serine/threonine-protein kinase TOR	Q9FR53
VPS15	Serine/threonine-protein kinase VPS15	Q9M0E5
VPS34	Phosphatidylinositol 3-kinase VPS34	P42339
KIN10	SNF1-related protein kinase catalytic subunit alpha KIN10	Q38997
KIN11	SNF1-related protein kinase catalytic subunit alpha KIN11	P92958
SNF4	Sucrose nonfermenting 4-like protein	Q944A6
GAPC2	Glyceraldehyde-3-phosphate dehydrogenase GAPC2, cytosolic	Q9FX54
GAPA-2	Glyceraldehyde-3-phosphate dehydrogenase GAPA2, chloroplastic	Q9LPW0
GAPCP2	Glyceraldehyde-3-phosphate dehydrogenase GAPC2, cytosolic	Q9FX54
PRC3	Proteasome subunit alpha type-2-A	O23708
RPT5B	26S proteasome regulatory subunit 6A homolog B	O04019
RPN3A	26S proteasome non-ATPase regulatory subunit 3 homolog A	Q9LNU4
PBC1	Proteasome subunit beta type-3-A	Q9XI05
RPN2B	26S proteasome non-ATPase regulatory subunit 1 homolog B	Q9MAT0
RPL10AA	Large ribosomal subunit protein uL1z	Q8VZB9
RPL10A	Large ribosomal subunit protein uL16z	Q93VT9
RPS27AA	Ubiquitin-ribosomal protein eS31z fusion protein	P59271
RACK1A	Small ribosomal subunit protein RACK1z	O24456
RPL6A	Large ribosomal subunit protein eL6z	Q9FZ76
RPL19A	Large ribosomal subunit protein eL19x	Q9SRX2
RPP1A	Large ribosomal subunit protein P1w	Q8LCW9
RIG	Small ribosomal subunit protein uS19u	Q08112
RPL21A	Large ribosomal subunit protein eL21z/eL21y	Q43291
RPL27AB	Large ribosomal subunit protein uL15y	Q9LR33
RPL34A	Large ribosomal subunit protein eL34z	Q42351
PFL	Small ribosomal subunit protein uS13z/uS13y/uS13x	P34788
RPS12A	Small ribosomal subunit protein eS12z	Q9S9P1
BMS1	P-loop containing nucleoside triphosphate hydrolase superfamily protein	F4IDR3
WDR	Uncharacterized protein At1g15425	Q8L403
PWP2	Periodic tryptophan protein 2	Q8VYZ5

## Data Availability

Not applicable.

## Supplementary Materials

The following supporting information can be downloaded at: https://www.mdpi.com/article/10.3390/life13102024/s1, Figure S1: Visualization of S-nitrosylated proteins with Western blot. Protein bands that were excised from the gel and subjected to in-gel trypsin cleavage for protein identification are marked on the blot; Figure S2: Functional annotation of 298 identified proteins; Table S1: Parameters of the nano-HPLC separation method employed in the nano-LC-QqTOF-MS-based proteomics experiments; Table S2: Instrument settings applied for ESI-QqTOF-MS DDA experiments employed in the nano-LC-QqTOF-MS-based proteomics experiments; Table S3: PEAKS Studio 10.6 parameters for database search settings; Table S4: Peptides, proteins, and protein groups identified in *Triticum aestivum*; Table S5: Functional classes of proteins identified in *Triticum aestivum*; Table S6: Localization of proteins identified in *Triticum aestivum*; Table S7: Hypothetical S-nitrosylation sites of identified proteins predicted with three programs; Table S8: Potential S-nitrosylation sites of autophagic proteins; Table S9: PPI of identified proteins, ATG proteins, and predicted functional partners.
