# Supplementary material for: S-Nitrosylated Proteins Involved in Autophagy in Triticum aestivum Roots: A Bottom-Up Proteomics Approach and In Silico Predictive Algorithms
Source: Life (Basel). 2023 Oct 8;13(10):2024. doi: 10.3390/life13102024 (PMC10608115; doi:10.3390/life13102024)
Supplement: Supplementary file 1 [file life-13-02024-s001.zip › Figures S1-S2.pdf]

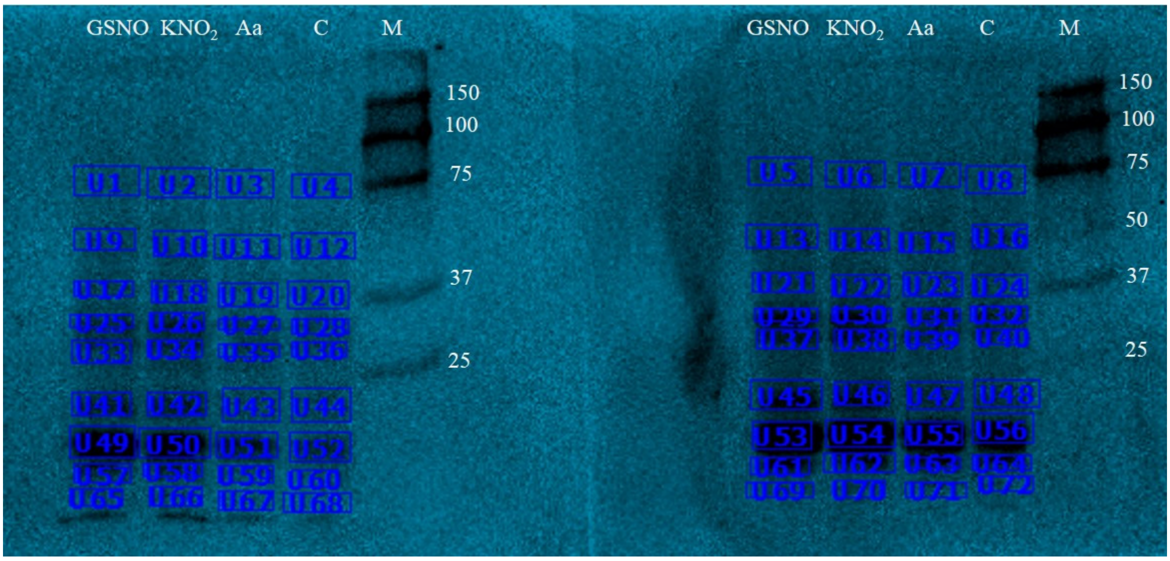

Figure S1. Visualization of S-nitrosylated proteins by Western blot. Protein bands that were excised from the gel and subjected to in-gel trypsin cleavage for protein identification are marked on the blot.

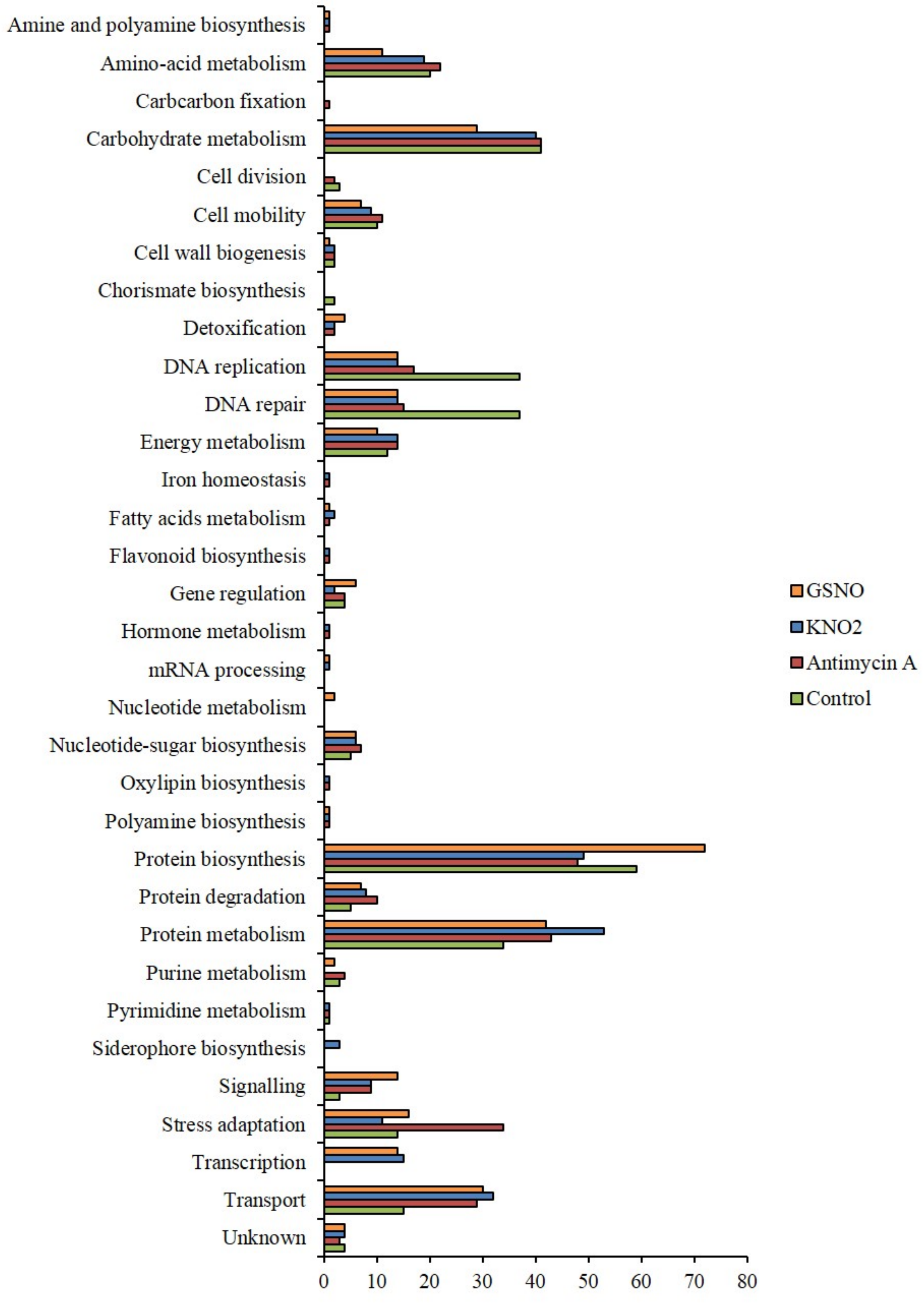

Figure S2. Functional annotation of 298 proteins identified proteins.
