# Supplementary material for: S-Nitrosylated Proteins Involved in Autophagy in Triticum aestivum Roots: A Bottom-Up Proteomics Approach and In Silico Predictive Algorithms
Source: Life (Basel). 2023 Oct 8;13(10):2024. doi: 10.3390/life13102024 (PMC10608115; doi:10.3390/life13102024)
Supplement: Supplementary file 1 [file life-13-02024-s001.zip › Tables S7-S8.pdf]

**Table S7. Hypothetical S-nitrosylation sites of identified proteins predicted by three programs.**

| Uniprot ID | Protein | Hypothetical S-nitrosylation sites |
|---|---|---|
| P46077 | 14-3-3-like protein GF14 phi | 106 |
| Q6W8Q2 | 1-Cys peroxiredoxin PER1 | 72 |
| Q9FIB6 | 26S proteasome non-ATPase regulatory subunit 12 homolog A | 397 |
| Q9LNU4 | 26S proteasome non-ATPase regulatory subunit 3 homolog A | 141 |
| Q9SEI4 | 26S proteasome regulatory subunit 6B homolog | 160 |
| P80602 | 2-Cys peroxiredoxin BAS1 chloroplastic (Fragment) | 64, 185 |
| Q8L8Y0 | 40S ribosomal protein S2-1 | 136, 236 |
| Q93VB8 | 40S ribosomal protein S2-2 | 136, 236 |
| P49688 | 40S ribosomal protein S2-3 | 137, 237 |
| Q9SCM3 | 40S ribosomal protein S2-4 | 128, 228 |
| Q9FJA6 | 40S ribosomal protein S3-3 | 97 |
| O48549 | 40S ribosomal protein S6-1 | 12, 56 |
| Q08682 | 40S ribosomal protein Sa-1 | 177 |
| Q1XIR9 | 4-hydroxy-7-methoxy-3-oxo-3 4-dihydro-2H-1 4-benzoxazin-2-yl glucoside beta-D-glucosidase 1a chloroplastic | 44 |
| D5MTF8 | 4-hydroxy-7-methoxy-3-oxo-3 4-dihydro-2H-1 4-benzoxazin-2-yl glucoside beta-D-glucosidase 1d chloroplastic | 44 |
| Q08770 | 60S ribosomal protein L10-2 | 72, 202 |
| P59230 | 60S ribosomal protein L10a-2 | 163 |
| Q9LHP1 | 60S ribosomal protein L7-4 | 182 |
| P49692 | 60S ribosomal protein L7a-1 | 194 |
| Q9LZH9 | 60S ribosomal protein L7a-2 | 193 |
| P46286 | 60S ribosomal protein L8-1 | 90 |
| Q9SH69 | 6-phosphogluconate dehydrogenase decarboxylating 1 chloroplastic | 377 |
| Q9FWA3 | 6-phosphogluconate dehydrogenase decarboxylating 2 | 375 |
| Q42560 | Aconitate hydratase 1 | 106 |
| Q9SIB9 | Aconitate hydratase 3 mitochondrial | 146, 533 |
| P53496 | Actin-11 OS=Arabidopsis thaliana | 12, 287 |
| P53497 | Actin-12 OS=Arabidopsis thaliana | 12, 287 |
| P53494 | Actin-4 OS=Arabidopsis thaliana | 12, 287 |
| Q43199 | Adenine phosphoribosyltransferase 1 | 153 |
| P32112 | Adenosylhomocysteinase | 268, 346 |
| O24396 | Adenylosuccinate synthetase chloroplastic (Fragment) | 188 |
| P31167 | ADP ATP carrier protein 1 mitochondrial | 130, 256 |
| Q41629 | ADP ATP carrier protein 1 mitochondrial | 81, 206 |
| P12862 | ATP synthase subunit alpha mitochondrial | 390 |
| P83483 | ATP synthase subunit beta-1 mitochondrial | 87 |
| P83484 | ATP synthase subunit beta-2 mitochondrial | 87 |
| Q9C5A9 | ATP synthase subunit beta-3 mitochondrial | 44, 90 |
| Q9SGY2 | ATP-citrate synthase alpha chain protein 1 | 98, 323 |
| O22718 | ATP-citrate synthase alpha chain protein 2 | 98, 323 |
| O80526 | ATP-citrate synthase alpha chain protein 3 | 98, 163 |
| P25696 | Bifunctional enolase 2/transcriptional activator | 346 |
| P38076 | Cysteine synthase OS=Triticum aestivum | 32 |
| A8MS68 | Dihydrolipoyl dehydrogenase 1 chloroplastic | 400, 441 |
| Q96327 | ERBB-3 binding protein 1 | 51, 82, 178 |
| Q9LF98 | Fructose-bisphosphate aldolase 8 cytosolic | 68 |
| O04834 | GTP-binding protein SAR1A | 173 |
| P22953 | Heat shock 70 kDa protein 1 | 319, 609 |
| Q9S7C0 | Heat shock 70 kDa protein 14 | 268, 781 |
| F4HQD4 | Heat shock 70 kDa protein 15 | 268 |
| O65719 | Heat shock 70 kDa protein 3 | 319, 326, 609 |
| Q39043 | Heat shock 70 kDa protein BIP2 | 298, 635 |

| | | |
|---|---|---|
| Q9ZP06 | Malate dehydrogenase 1 mitochondrial | 130 |
| Q95748 | NADH dehydrogenase [ubiquinone] iron-sulfur protein 3 | 60 |
| P93596 | Obtusifoliol 14-alpha demethylase (Fragment) | 305 |
| P26759 | Oxalate oxidase GF-3.8 OS=Triticum aestivum | 33 |
| Q9LNE3 | Probable fructokinase-2 | 300 |
| Q9LNE4 | Probable fructokinase-3 | 299 |
| Q9M1B9 | Probable fructokinase-4 | 299 |
| O82616 | Probable fructokinase-5 OS=Arabidopsis thaliana | 45, 84, 295 |
| O23715 | Proteasome subunit alpha type-3 | 217 |
| Q7DLS1 | Proteasome subunit beta type-7-B | 215 |
| Q9FEF8 | rRNA 2'-O-methyltransferase fibrillarin 1 | 252 |
| Q94AH9 | rRNA 2'-O-methyltransferase fibrillarin 2 | 263 |
| O23254 | Serine hydroxymethyltransferase 4 | 187, 367 |
| Q9SVM4 | Serine hydroxymethyltransferase 5 | 324 |
| O48661 | Spermidine synthase 2 | 43, 120 |
| Q940P8 | T-complex protein 1 subunit beta | 131, 469 |
| P48491 | Triosephosphate isomerase cytosolic | 13, 127 |
| Q9ZRB7 | Tubulin alpha chain OS=Triticum aestivum | 118, 376 |
| Q9ZRB2 | Tubulin beta-1 chain OS=Triticum aestivum | 12 |
| Q9ZRA8 | Tubulin beta-5 chain OS=Triticum aestivum | 12 |
| P59271 | Ubiquitin-40S ribosomal protein S27a-1 | 126 |
| P59232 | Ubiquitin-40S ribosomal protein S27a-2 | 126 |
| P59233 | Ubiquitin-40S ribosomal protein S27a-3 | 126 |
| Q9ZUY6 | UDP-D-apiose/UDP-D-xylose synthase 1 | 173, 187 |
| Q9SGE0 | UDP-D-apiose/UDP-D-xylose synthase 2 | 173, 187 |
| P57751 | UTP--glucose-1-phosphate uridylyltransferase 1 | 96 |
| Q9M9P3 | UTP--glucose-1-phosphate uridylyltransferase 2 | 95 |

**Table S8.** Potential S-nitrosylation sites of autophagic proteins.

| Uniprot ID | Protein | Hypothetical sites of S-nitrosylation |
|---|---|---|
| C0LP24 | Cysteine protease (ATG4) | 48 |
| U3Q008 | Cysteine protease (ATG4b) | 48 |
| U3PWQ4 | Cysteine protease (ATG4a) | 47 |
| U3PWS4 | Autophagy-related protein 16a | 391 |
| U3Q035 | Autophagy-related protein 16b | 391 |
