# Supplementary material for: S-Nitrosylated Proteins Involved in Autophagy in Triticum aestivum Roots: A Bottom-Up Proteomics Approach and In Silico Predictive Algorithms
Source: Life (Basel). 2023 Oct 8;13(10):2024. doi: 10.3390/life13102024 (PMC10608115; doi:10.3390/life13102024)
Supplement: Supplementary file 1 [file life-13-02024-s001.zip › Tabless S1-S3.pdf]

Table S1. Parameters of the nanoHPLC separation method employed in the nanoLC-QqTOF-MS-based proteomics experiments.

Parameter	Settings	
	Method parameters	
Injection volume	2 µL	
Injection mode	sample loading pressure 217.5 bar	
Column temperature	45⁰C	
	Eluents	
Solvent A	0.1% (v/v) aq. formic acid	
Solvent B	0.1% (v/v) formic acid in acetonitrile	
Elution regimen	Time (min)	% B
	0	2
	20	40
	20,5	85
	35,9	85
	37,9	2
	40	2
Trap Column	Thermo Trap Cartridge 5mm	
Volume	0.148 µL	
Equilibration pressure	217.5 bar	
Estimated equilibration time	0.65 min	
Equilibration volume (×10)	1.48 µL	
Separation Column	Bruker FORTY	
Volume	0.742 µL	
Equilibration pressure	600.0 bar	
Estimated equilibration time	7.91 min	
Equilibration volume (×4)	2.97 µL	

Table S2. Instrument settings applied for ESI-QqTOF-MS DDA experiments employed in the nanoHPLC-QqTOF-MS-based proteomics experiments.

Parameter	Settings
MS conditions	
Ionization mode	Positive
Mass to charge ratio (*m/z*) range	150 – 2200
Spectra rate	2 Hz
End plate offset	500 V
Capillary voltage	4500 V
Nebulizer	1.5 bar
Dry temperature	200°C
Dry gas	2.0 l/min
MS/MS conditions	
Scan mode	Auto MS/MS
Fragmentation type	Collision-induced dissociation
Isolation width	2 – 3
MS/MS spectra acquisition	8 – 32 Hz
Threshold (per 1000 sum.)	250 cts
Cycle time	3 sec
Collision energy	from 23 eV (m/z 300) to 65 eV (m/z 1300)
Scan mode	Auto MS/MS

Table S3. PEAKS Studio 10.6 parameters for database search settings.

Database search settings	
Analysis program	PEAKS Studio 10.6 build 20201221
Parent mass error tolerance:	10.0 ppm
Fragment mass error tolerance:	0.05 Da
Precursor mass search type:	Monoisotopic
Protease	Trypsin
Missed cleavage sites	2
FDR	2
Fixed modifications:	Carbamidomethylation: 57.02
Variable modifications	Oxidation (M): 15.99
	Acetylation (Protein N-term): 42.01
	Deamidation (NQ): 0.98
	S-nitrosylation: 28.99
Max variable PTM per peptide	2
Filter charge	1 – 7
